# A cytogenetical study on *Economidichthys pygmaeus* Holly, 1929 (Pisces, Gobiidae), an endemic freshwater goby from Western Greece

**DOI:** 10.3897/CompCytogen.v5i5.2015

**Published:** 2011-12-22

**Authors:** Massimiliano Rampin, Christos Gkenas, Stefano Malavasi, Angelo Libertini

**Affiliations:** 1 Centro de Biologia Ambiental, Faculdade de Ciências - Universidade de Lisboa – Portugal, Campo Grande - 1749-016 Lisboa - Portugal; 2 Department of Biological Applications and Technology, University of Ioannina, Ioannina, PC 45110, Greece; 3 Department of Environmental Sciences, Informatics and Statistics, Ca’ Foscari University of Venice, Castello, 2737/B, 30122 Venice, Italy; 4 CNR-Institute of Marine Sciences, Castello, 1364/A, 30122 Venice, Italy

**Keywords:** Goby fish, karyotype, fluorescent *in situ* hybridization, genome size

## Abstract

A cytogenetic study was carried out on the chromosomes and the nuclear DNA content of the freshwater goby *Economidichthys pygmaeus* (Pisces, Gobiidae). The species is characterized by a 2n=46 karyotype consisting of 12 submetacentric and 11 subtelocentric chromosome pairs (NF=70). Major (45S) rDNA genes are terminal-centromeric located on the short arm of a single medium-small sized submetacentric pairas assessed by *in situ* hybridization, CMA_3 _staining, and Ag-NOR banding. The haploid (C-value) nuclear DNA content is 0.93±0.003 picograms. The cytogenetical data of *Economidichthys pygmaeus* were compared with those ones already available for other related gobies.

## Introduction

*Economidichthys pygmaeus* is an endemic small-bodied, short-lived species restricted to the north and western part of Greece (Miller 1990). Specifically, it is distributed in the Thiamis, Louros, Arahcthos and Achelloos Rivers and in Lakes Trichonis, Lyssimachia, Ziros and Ozeros. Recently it has been recorded in Lake Pamvotis where it was introduced probably from River Louros and/or Kalamas (Thyamis) (Leonardos et al. 2008). The species appears to be extinct on Lefkas Island (Economidis 1991) and is protected under Greek law No. 67/1981. Greece’s updated edition of the Red Book of Endangered Species (2009) has evaluated its conservation status and it is considered now a ‘least concerned species’, without facing any critical dangers (Crivelli 2006, Economidis 2009). Despite the importance of this species in terms of conservation, information on many aspects of its biology, ecology and behaviour are lacking in the current literature, and no data on karyotype are available at present (Miller 1990). In the aim of promoting the conservation of this species a set of investigations was carried out in order to better understand its biology and life-history. In such research frame the present cytogenetical study is included.

## Materials and methods

Ten adult males and five females of *Economidichthys pygmaeus* were collected from Lake Pamvotis (Ioannina, NW Greece) and used for this study. Animals were injected with colchicine and were killed with an overdose of MS222. After that, the fish were sacrificed and chromosome preparations were obtained from spleen and testis by using the conventional air-drying technique. Chromosome plates were conventionally stained with Giemsa and eventually re-stained with silver nitrate (Howell and Black 1980) to get an Ag-NOR banding. Chromosome classification follows Levan et al. (1964). Mapping of rDNA major complex genes were performed by fluorescent *in situ* hybridization (FISH) with the pDm238 probe (Roiha et al*.* 1981) containing the 18S-5.8S-28S gene cluster and intergenic spacers of the fruit fly *Drosophila melanogaster* Meigen, 1830, according to the procedure in Libertini et al. (2008). Plates previously analysed by FISH were sequentially stained with chromomycin A_3_ (CMA_3_) (Schweizer 1976). Genome size (GS) was assessed by flow cytometry on peripheral erythrocytes, according to the method in Libertini et al. (2003).

## Results

**Figure 1.**
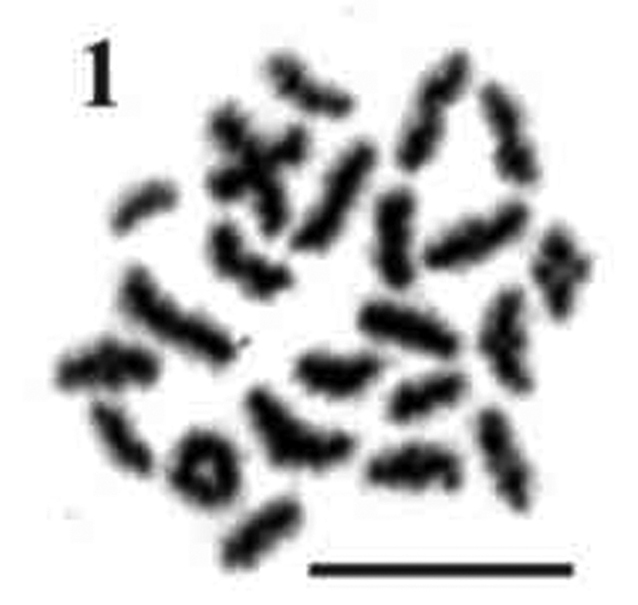
*Economidichthys pygmaeus* first spermatocyte metaphase. Bar = 10μm

**Figure 2.**
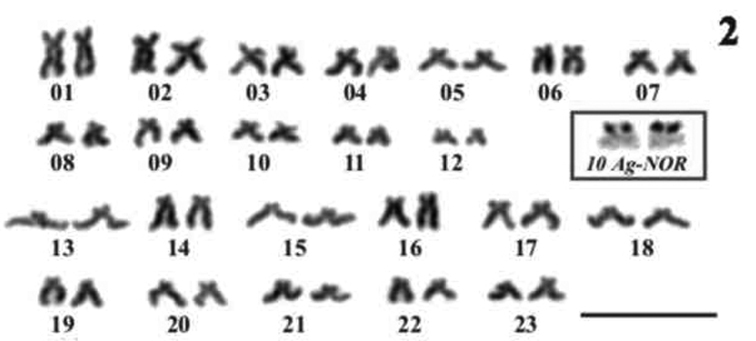
*Economidichthys pygmaeus* karyotype. Pairs 1–12 submetacentrics; pairs 13-23 subtelocentrcs. Inset Ag-NOR staining of pair 10. Bar = 10µm.

**Figure 3.**
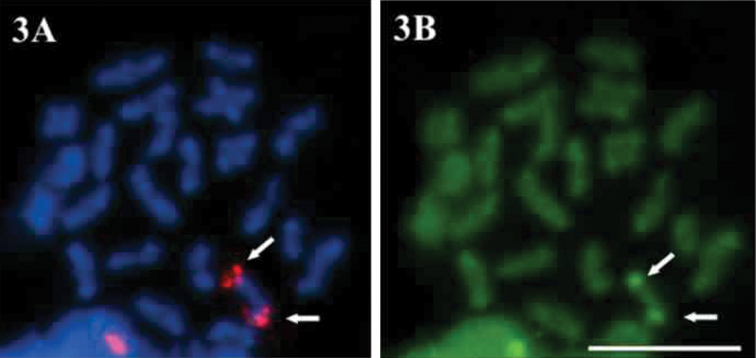
**A–B** *Economidichthys pygmaeus* first spermatocyte metaphase **A** FISH with a rDNA major complex probe **B **Sequential staining with CMA_3_.

**Figure 4.**
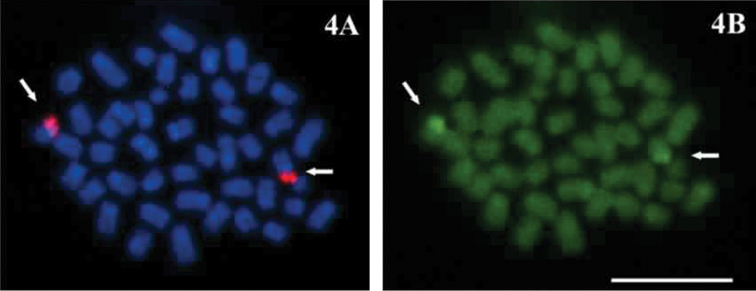
**A–B** *Economidichthys pygmaeus* mitotic metaphase **A** FISH with rDNA major complex probe **B** Sequential staining with CMA_3_. Bar = 10µm.

The haploid n=23 and the diploid 2n=46 chromosome numbers were determined for *Economidichthys pygmaeus* (Fig. 1 and Fig. 2) from the counts of 50 and 94 plates, respectively. All the analysed specimens, regardless of sex, shared the same 2n=46 karyotype (Fig. 2), composed of 24 submetacentric (Fig. 2, pairs 1-12) and 22 subtelocentric (Fig. 2, pairs 13-23) chromosomes. Therefore, the fundamental number of chromosome arms (NF) is 70. Ag-NOR banding (Howell and Black 1980) showed a terminal-centromeric location (following the scheme in Caputo 1998) of the active nucleolar organiser regions (Fig. 2, blue inset) on the short arm in a medium-small sized submetacentric pair (Fig. 1, pair 10). FISH results from first spermatocyte metaphase bivalents (Fig. 3A) and mitotic chromosomes (Fig. 4A) confirmed the previous observations with Ag-NOR banding. In fact, a single bivalent showed hybridization signals with the NORs probe on both ends (Fig. 3A, arrows) in the spermatocyte plates, while in mitotic plates a couple of chromosomes showed rDNA major complex FISH signals on the short arm (Fig. 4A, arrows). CMA_3 _produced overlapping bright signals in the same location of hybridization signals (Figs 3B and 4B, see arrows), indicating that rDNA major complex gene sequences contain GC-rich DNA. Through flow cytometric essay the GS (haploid C-value) of *Economidichthys pygmaeus* was evaluated as 0.93±0.003 picograms.

## Discussion

About one hundred species of Gobiidae have been analysed cytogenetically and show great karyotypic diversity. Diploid number varies from 30 to 62 and variation in chromosome morphology is also wide (NF=40-98). High intraspecific chromosomal variability has been detected in some species (Galetti et al. 2000, and reference therein). In the comparison with the other gobies *Economidichthys pygmaeus* is characterized by the most common chromosome number (2n=46) and by an unusual karyotype composed exclusively by submetacentric and subtelocentric elements. The resulting NF=70 is intermediate among NF values of Gobiidae (Klinkhardt et al. 1995, and reference therein). The GS of *Economidichthys pygmaeus* (0.93 pg) is also intermediate among the GS values of the Gobiidae (range 0.42-1.68 pg) (Animal Genome Size Database 2009). A single pair of medium-small chromosomes bearing a GC-rich NOR on a terminal-centromeric zone in the short arm is the most common location of major rDNA genes in Gobiidae, being shared by the genera *Gobius* Linnaeus, 1758, *Pomatoschistus* Gill, 1863, *Knipowitschia* Iljin, 1927 and *Economidichthys* Bianco, Bullock, Miller et Roubal, 1987 (Caputo 1998; present paper; A. Libertini unpublished data), and this location represents probably the plesiomorphic character state (Caputo 1998). The genus *Economidichthys* along with the genera *Pomatoschistus*, *Gobiusculus* Duncker, 1928, and *Knipowitschia* were gathered in the so-called sand goby group (McKay and Miller 1997, Huyse et al. 2004). The sand gobies were clustered as a monophyletic group on morphological (McKay and Miller 1997), molecular (Huyse et al. 2004) and behavioural (Malavasi et al. 2008) grounds. Monophyly of sand gobies is also suggested by the sharing of common, probably plesiomorphic, cytogenetical characters: the chromosome number 2n=46 is present in all the four genera (Klinkhardt et al. 1995, Animal Genome Size Database 2009, present paper), the common NOR location in a terminal-centromeric zone in the short arm in a single submetacentric pair for the genera *Pomatoschistus*, *Knipowitschia* and *Economidichthys* so far studied (Caputo 1998, present paper, Libertini unpublished data), and similar GS values in a narrow range around 1 pg (0.91–1.04 pg) (Animal Genome Size Database 2009, present paper). The wide variability of karyotype formula and NF vsa general constancy of chromosome numbers indicate that non-Robertsonian mechanisms of chromosome rearrangements were more frequently involved in karyotype evolution of the sand gobies.

## References

[B1] Animal Genome Size Database (2009) http://www.genomesize.com

[B2] Caputo V (1998) Nucleolar organizer (NOR) location and cytotaxonomic implications in six species of gobiid fishes (Perciformes, Gobiidae). Italian Journal of Zoology 65: 93-99 doi: 10.1080/11250009809386729

[B3] Crivelli AJ (2006)*Economidichthys pygmaeus*. In: IUCN 2008. 2008 IUCN Red List of Threatened Species. [www.iucnredlist.org accessed on 27 January 2009]

[B4] Economidis PS (1991) Check list of freshwater fishes of Greece: recent status of threats and protection. Hellenic Society for Protection of Nature, Athens, 48 pp.

[B5] Economidis PS (2009) Freshwater fishes of Greece. In: Legakis A and Maragkou P (Eds). Red book of endangered species in Greece (in Greek).Hellenic Zoological Society, Athens, Greece: 86-159

[B6] Galetti PM Jr Aguilar CT Molina WF (2000) An overview of marine fish cytogenetics. Hydrobiologia 420: 55-62 doi: 10.1023/A:1003977418900

[B7] Howell WM Black DA (1980) Controlled silver-staining of nucleolus organizer regions with a protective colloidal developer: a 1-step method. Cellular and Molecular Life Sciences 36: 1014-1015 doi: 10.1007/BF01953855 10.1007/BF01953855 6160049

[B8] Huyse T Van Houdt J Volckaert FAM (2004) Paleoclimatic history and vicariant speciation in the “sand goby” group (Gobiidae, Teleostei). Molecular Phylogenetics and Evolution 32: 324-336 doi: 10.1016/j.ympev.2003.11.007 15186817 10.1016/j.ympev.2003.11.007

[B9] Klinkhardt M Tesche M Greven H (1995) Database of fish chromosomes. Westarp Wissenschaften, Magdeburg, 237 pp.

[B10] Leonardos ID Kagalou I Tsoumani M Economidis PS (2008) Fish fauna in a protected Greek lake: biodiversity, introduced fish species over a 80-year period and their impacts on the ecosystem. Ecology of Freshwater Fish 17: 165-173 doi: 10.1111/j.1600-0633.2007.00268.x

[B11] Levan A Fredga K Sandberg AA (1964) Nomenclature for centromeric position on chromosomes. Hereditas 52: 201-220 doi: 10.1111/j.1601-5223.1964.tb01953.x

[B12] Libertini A Mandrioli M Colomba MS Bertotto D Francescon A Vitturi R (2003) A cytogenetic study of the common sole, *Solea solea*, from the Northern Adriatic Sea. Chromosome Science 6: 63-66

[B13] Libertini A Sola L Rampin M Rossi AR Iijima K Ueda T (2008) Classical and molecular cytogenetic characterization of allochthonous European bitterling *Rhodeus amarus* (Cyprinidae, Acheilognathinae) from Northern Italy. Genes and Genetic Systems 83 (5): 417-422 doi: 10.1266/ggs.83.417 19168992 10.1266/ggs.83.417

[B14] Malavasi S Collatuzzo S Torricelli P (2008) Interspecific variation of acoustic signals in Mediterranean gobies (Perciformes, Gobiidae): comparative analysis and evolutionary outlook. Biological Journal of the Linnean Society 93 (4): 763-778 doi: 10.1111/j.1095-8312.2008.00947.x

[B15] McKay SI Miller PJ (1997) The affinities of European sand gobies (Teleostei: Gobiidae). Journal of Natural History 31: 1457-1482 doi: 10.1516/T834-3854-181N-8P85

[B16] Miller PJ (1990) The endurance of endemism: the Mediterranean freshwater gobies and their prospects for survival. Journal of Fish Biology 37 (Suppl. A):145–156. doi: 10.1111/j.1095-8649.1990.tb05030.x

[B17] Roiha H Miller JR Woods LC Glover DM (1981) Arrangements and rearrengements of sequences flanking the two types of rDNA insertion in *D. melanogaster*. Nature 290: 49-53 doi: 10.1038/290749a0 6783966 10.1038/290749a0

[B18] Schweizer D (1976) Reverse fluorescent chromosome banding with chromomycin and DAPI. Chromosoma 58: 307-324 doi: 10.1007/BF00292840 137107 10.1007/BF00292840

